# Role of age, Rho‐kinase 2 expression, and G protein‐mediated signaling in the myogenic response in mouse small mesenteric arteries

**DOI:** 10.14814/phy2.13863

**Published:** 2018-09-10

**Authors:** Karl Björling, Philomeena D. Joseph, Kristian Egebjerg, Max Salomonsson, Jakob L. Hansen, Trine P. Ludvigsen, Lars J. Jensen

**Affiliations:** ^1^ Department of Veterinary and Animal Sciences Faculty of Health and Medical Sciences University of Copenhagen Frederiksberg C Copenhagen Denmark; ^2^ Department of Biomedical Sciences Faculty of Health and Medical Sciences University of Copenhagen Copenhagen N Denmark; ^3^ Department of Internal Medicine Trelleborg Hospital Trelleborg Sweden; ^4^ Cardiovascular Research Novo Nordisk A/S Måløv Denmark

**Keywords:** Aging, blood pressure, Göttingen minipig, G*α*12, G*α*q/11, hypertension, mouse, myogenic tone, ROCK2

## Abstract

The myogenic response (MR) and myogenic tone (MT) in resistance vessels is crucial for maintaining peripheral vascular resistance and blood flow autoregulation. Development of MT involves G protein‐coupled receptors, and may be affected by aging. Aims: (1) to estimate the mesenteric blood flow in myogenically active small mesenteric arteries; (2) to investigate the signaling from G_*α*q/11_ and/or G_*α*12_ activation to MT development; (3) to investigate the role of Rho‐kinase 2 and aging on MT in mesenteric resistance arteries. Methods: we used pressure myography, quantitative real‐time PCR, and immunolocalization to study small (<200 *μ*m) mesenteric arteries (SMA) from young, mature adult, and middle aged mice. Results: Poiseuille flow calculations indicated autoregulation of blood flow at 60−120 mm Hg arterial pressure. G_*α*q/11_ and G_*α*12_ were abundantly expressed at the mRNA and protein levels in SMA. The G_*α*q/11_ inhibitor YM‐254890 suppressed MT development, and the Phosholipase C inhibitors U73122 and ET‐18‐OCH3 robustly inhibited it. We found an age‐dependent increase in ROCK2 mRNA expression, and in basal MT. The specific ROCK2 inhibitor KD025 robustly inhibited MT in SMAs in all mice with an age‐dependent variation in KD025 sensitivity. The inhibitory effect of KD025 was not prevented by the L‐type Ca^2+^ channel activator BayK 8644. KD025 reversibly inhibited MT and endothelin‐1 vasoconstriction in small pial arteries from Göttingen minipigs. Conclusions: MT development in SMAs occurs through a G_*α*q/11_/PLC/Ca^2+^‐dependent pathway, and is maintained via ROCK2‐mediated Ca^2+^ sensitization. Increased MT at mature adulthood can be explained by increased ROCK2 expression/activity.

## Introduction

The myogenic response is a property possessed by resistance arteries. It is defined as an increase in vascular tone that follows a raise in transmural pressure, or as a decrease in vascular tone that follows a decline in transmural pressure (Schubert and Mulvany 1999). The physiological importance is still not completely understood, but three important roles have previously been pointed out by others. The first, “basal vascular tone” gives other control mechanisms opportunity to both increase, through dilation, and decrease, through constriction, blood flow. The second function, “autoregulation of flow and pressure” means that the myogenic response plays a central role in the preservation of a steady perfusion of the vasculature during changes in blood pressure (Davis and Hill 1999). As a third aspect, Izzard and others discuss the issue that myogenic tone can protect vessels from vascular hypertrophy by lowering wall stress, which has been proposed to be the stimulus for hypertrophy (Izzard et al. 2005). Recent studies have provided evidence that the G protein‐coupled receptor (GPCR) mediated signaling is involved (Schnitzler et al. 2008; Schleifenbaum et al. 2014; Hui et al. 2015; Storch et al. 2015; Kauffenstein et al. 2016) but mechano‐activated ion channels (Welsh et al. 2002; Drummond et al. 2004; Earley et al. 2004; Bulley et al. 2012) and interactions between extracellular matrix proteins and membrane‐spanning integrins (Martinez‐Lemus et al. 2003, 2005; Jackson et al. 2010; Colinas et al. 2015) are also thought to contribute to the upstream sensing of pressure in the myogenic response. More detailed knowledge of the signaling pathways in the myogenic response is needed for a more complete understanding of the pathogenesis of hypertension and conditions that entail important risk factors for hypertension, such as: aging, abdominal obesity, diabetes, chronic mental stress, excess salt intake, high cholesterol, lack of physical exercise, or sleep apnea. An improved understanding is also necessary to be able to use the myogenic response as a potential target in pharmacological treatment (Hill et al. 2009). Finally, a complete understanding of the signaling components involved in the myogenic response requires that detailed investigations are carried out in individual vascular beds, as there might be differences between, for example, cerebral, skeletal muscle, coronary, renal, and mesenteric resistance vessels.

Aging is a factor linked to several cardiovascular complications such as essential (idiopathic) hypertension, renal disease, and cerebral blood flow complications, that is, stroke and vascular dementia. The mechanisms involved in aging‐induced hypertension are incompletely understood, but may involve changes in arterial compliance, endothelial function, production of reactive oxygen species, and renal function (Sawabe 2010; Bachschmid et al. 2013; Barton et al. 2016; Buford 2016; Pont and Alhawassi 2017). Increased total peripheral resistance contributes to aging‐induced hypertension due to endothelial dysfunction and hypercontractility of small resistance arteries (Toro et al. 2002; Yildiz 2007; El et al. 2012), but knowledge of the impact of aging on myogenic tone (MT) and autoregulation of blood flow mostly stems from the cerebral circulation. To this end, the cognitive decline often seen with aging may be correlated with deficient regulation of cerebral blood flow (Toth et al. 2017), and neurodegenerative diseases such as Alzheimer's Disease, Vascular Dementia and Parkinson's Disease have been linked with defective cerebral blood flow autoregulation (Vokatch et al. 2007; de la Torre 2012; Brickman et al. 2015). In a recent study, we found that aging was correlated with an increased responsiveness to norepinephrine as well as enhanced myogenic tone (Mikkelsen et al. 2016). This is consistent with a mechanism that involves signaling pathways downstream of GPCR activation. Another recent study showed that an age‐dependent increase in blood pressure is associated with procontractile activation of both G_*α*q/11_‐ and G_*α*12_‐mediated pathways (Wirth et al. 2016). G_*α*q/11_ and G_*α*12_ are upstream activators of the RhoA/Rho‐associated kinase (ROCK) pathway via several different Rho‐Guanine Exchange Factors (RhoGEFs) (Gohla et al. 2000; Booden et al. 2002; Chikumi et al. 2002; Vogt et al. 2003; Lutz et al. 2005). ROCK plays a central role in vascular smooth muscle contraction, via phosphorylation of the Myosin Light Chain Phosphatase (MLCP) subunit MYPT‐1, thereby inhibiting MLCP and causing Ca^2+^‐sensitization of the contractile filaments in vascular smooth muscle cells (VSMCs) (Somlyo and Somlyo 2003; Schubert et al. 2008; Cole and Welsh 2011). ROCK has been shown to contribute significantly to increased Ca^2+^ sensitization during the myogenic response in resistance arteries from the brain and from skeletal muscle (Bolz et al. 2003; Gokina et al. 2005; Jarajapu and Knot 2005; Johnson et al. 2009; Moreno‐Dominguez et al. 2013). It has moreover been shown that although both ROCK1 and ROCK2 isoforms are abundantly expressed in VSMC, ROCK2 is the predominant isoform to regulate VSMC contractility (Wang et al. 2009) and thus the most relevant isoform regarding the Ca^2+^ sensitization process during the myogenic response. In this study, we therefore sought to investigate the role of ROCK2 in the age‐dependent changes in myogenic tone.

Since it is not widely accepted that mesenteric resistance vessels have basal tone, we wanted to investigate whether the myogenic response that we have measured in mouse small mesenteric arteries (SMA) would in theory give rise to autoregulation of blood flow. Thus, the first aim of our study was to estimate the theoretical blood flow based on our measurements of the myogenic response in SMAs from young wild type mice. Furthermore, as there might be important differences between the mechanism of spontaneous myogenic tone development between different vascular beds, we wanted to investigate the signaling pathways involved in young wild type mouse mesenteric arteries, because these are often used in comparison with knock‐out mice. Thus, our second aim was to use a pharmacological approach to define the signaling network(s) leading from GPCR activation to myogenic tone development in mouse SMAs. Finally, our third aim was to test the putative role of ROCK2 in the aging‐induced increase in myogenic tone, which we had found in a previous study (Mikkelsen et al. 2016).

## Methods

### Mouse small artery preparation and pressure myography

Male wild type C57BL6/J mice in three age groups (2–3 months; 6–7 months; 13–14 months) were housed, handled, and eventually sacrificed by cervical dislocation according to permission obtained from The Animal Experiment Inspectorate, The Danish Ministry of Justice. Third order SMAs were quickly dissected free from excess fat and connective tissue, and mounted in a pressure myograph system (Model 120 CP, DMT A/S, Århus, Denmark) as previously described in detail (Björling et al. 2013; Mikkelsen et al. 2016). To relax and protect the vessels, a nominal Ca^2+^‐free HEPES‐buffered PSS (Mounting buffer) was used while mounting the vessels between two fire‐polished glass pipettes of matching resistances (80 *μ*m outer diameter) and securing them with two 11‐0 nylon suture knots at both ends. After mounting, the vessels were superfused with Krebs’ buffer (flow rate ~2.5 mL/min) that was equilibrated with 95% O_2_/5% CO_2_ (pH 7.4) at 37°C. The pressure was now slowly raised to 120 mm Hg and the vessels were stretched to their presumed in vivo length, as judged by visual inspection and by assuring that the longitudinal force measurement did not exceed 0.35 mN at any time. To exclude leaks, it was ensured that the pressure at both ends of the artery did not differ by more than 1–2 mm Hg. The luminal perfusate was Krebs’ buffer to which 1% low‐endotoxin bovine serum albumin was added, and experiments were performed under no‐flow conditions. The vitality of the vessel preparations was initially tested at 40 mm Hg intraluminal pressure by exposure to High‐KCl (75 mmol/L; K75) followed by a submaximal concentration of norepinephrine (1 *μ*mol L^−1^; NE). Vessel preparations that contained leaks or did not respond within 30 sec with a uniform constriction of at least 50% to K75 and 20% to NE were discarded. The pressure was now maintained at 80 mm Hg for 30–40 min to allow basal myogenic tone to be established. At this point the first (active) pressure curve was constructed by measuring the diameter at incremental pressure steps of 20 mm Hg from 20 to 120 mm Hg. Both the lumen and external (vessel) diameters were measured at steady‐state (4–5 min after each pressure step). After the active pressure curve, the response to K75 was measured at 40 mm Hg. A pharmacological test drug was now added to the myograph chamber dissolved in Krebs’ buffer, and allowed to incubate for 10 min before the second (drug) pressure curve was constructed in exactly the same manner as the active curve. After this pressure curve was completed, the response to K75 was measured in the presence of the drug at a pressure of 40 mm Hg. This K75 response (with drug) was compared with the K75 response (without drug) obtained immediately before the second pressure curve. If the peak K75 response was inhibited in the presence of drug, we performed a new experiment using a lower drug concentration until no such inhibitory effect on the K75 response was seen. This precaution was taken to rule out unspecific drug effects on the pressure‐diameter curves, which were not due to inhibition of myogenic tone. Finally, the vessel preparation was superfused with Ca^2+^‐free Krebs’ buffer containing EGTA (2 mmol L^−1^) for 20 min, and the passive pressure curve was constructed in the same pressure range as for the two‐first pressure curves.

### Minipig pial artery preparation

Three female Göttingen minipigs (age 54 ± 7 weeks and body weight 26.4 ± 1.2 kg at sacrifice) were obtained from Ellegaard Göttingen Minipigs A/S (Dalmose, Denmark). The minipigs were brought to surgical anesthesia by *i.m*. injection of Zoletil^®^50 mixture as previously described (Pedersen et al. 2014) and bled from the axillae for euthanasia. The animals had prior to euthanasia been subjected to different pharmacological studies (not further described here), and were used after washout of the tested substances was achieved. The animals were single‐housed under a normal day/night cycle with a relative humidity of 50–70% and a room temperature of 22–24°C. They were fed standard minipig chow (Special Diet Services, UK and France: Standard minipig diet, Mini‐Pig), according to the breeder's recommendations and with free access to water and bedding material. After euthanasia, and opening of the skull using a bandsaw, the brain was gently lifted out of the cranium. The middle cerebral arteries and their attached smaller pial arteries and arterioles were subsequently removed from the brain surface by blunt dissection. Using sharpened ophthalmic scissors and forceps, the small pial arteries were carefully removed and mounted in the pressure myograph as decribed for mouse SMAs above. The study was approved by the Animal Experiment Inspectorate, The Danish Ministry of Justice.

### Theoretical blood flow calculations

Data from active pressure‐diameter curves obtained in mouse SMAs was inserted in the Hagen‐Poiseuille flow equation (eq. (1)) modified with an expression of relative viscosity (eq. (2)) (Pries et al. 1992). To fulfill the requirements of the Hagen‐Poiseuille equation we considered each vessel as a straight, rigid, cylindrical tube and with constant radius within the segment analyzed. The velocity profile of the flow was considered parabolic and the estimated flow was regarded as applicable only to a short moment in time during which the flow can be seen as stable instead of pulsatile. The driving pressure was considered constant due to the principle that specific locations in the mesenteric arcade at any moment in time holds a specific fraction of the systemic blood pressure, even when this is changing. This was previously described in studies where the pressure was measured in different locations in the mesenteric arcade in conscious, freely moving rats (Fenger‐Gron et al. 1995). The equation for relative viscosity was used because blood viscosity decreases in a nonlinear fashion at lumen diameters smaller than ~300 *μ*m. Even though this equation is mostly based on studies from human material, which has been described to have about 19% higher viscosity than mice (Windberger et al. 2003), it was considered an advantage.

(1) $$Q=\Delta P\pi r^{4}/8\eta l$$

where *Q *= flow; Δ*P* = pressure gradient; *r *= vessel radius, *η *= viscosity, and *l *= vessel length. And

(2) $$\eta_{rel0.45}=220e^{-1.3D}+3.2-2.44e^{-0.06D^{0.645}}$$

where *η*_rel0.45_ = relative viscosity at hematocrit level 0.45, and *D *= lumen diameter.

### Quantitative real‐time PCR (QPCR)

Primer pairs (forward and reverse) for each gene were designed through Primer Blast. The following primer set was used for mRNA quantification of Gna11 (G_*α*q/11_): Forward − GAGGACCGCGACGATGAC; Reverse − CCTGCATGGCGGTAAAGATG (278 bp; Acc. No. NM_010301.3). For Gna12 determination (G_*α*12_) we used: Forward − CGAGTTCCAGCTGGGTGAAT; Reverse − CATACTCGCTCGAGGACACC (260 bp; Acc. No. NM_010302.2). For Rock2 determination (ROCK2) we used: Forward − AAAACTGTGATCCCAAGGGAAG; Reverse − CACATGAACTGAGCAAAGCCC (85 bp; Acc. No. NM_009072.2). For quantification of the reference gene Actb (*β*‐actin) we used: Forward − AGCCATGTACGTAGCCATCC; Reverse − CTCTCAGCTGTGGTGGTGAA (228 bp; Acc. No. NM_007393.3). SMAs, middle cerebral arteries (MCA), and renal arteries (RA) were freshly dissected from young mice and used for total RNA extraction using a TissueLyzer apparatus and RNEasy Micro kit (Qiagen, Hildenberg, Germany). Mouse brain was used as a calibrator sample. cDNA (RT^+^ and RT^–^) made from the freshly extracted RNA was prepared using a Promega kit according to the manufacturer's information. QPCR was performed using a LightCycler 480^®^ apparatus (Roche; Roche Diagnostics A/S, Hvidovre, Denmark) with PCR Mastermix containing SYBR^®^ Green, primer pairs, and RT^+^ or RT^−^ samples. The QPCR protocol was annealing at 60°C for 10 sec, and elongation at 72°C for 20 sec. The housekeeping gene Actb (*β*‐Actin) was used as reference gene as its expression level (*C*_T_ values) did not vary significantly between the different samples. Standard curves for each primer set were prepared using dilution series [1:8–1:1024] of cDNA from the calibrator sample, and yielded efficiencies in the range of 1.8–2.2 and Slopes in the range of −3.3 to −3.6. Melting curves contained only single peaks. For each primer set the product size was of the expected size, and there were no amplifications in negative control samples (H_2_O; RT^−^). QPCR data were reported as the normalized relative ratio, 2^−ΔΔCT^, where ΔCT = CT (target) – CT (ActB), and ΔΔCT = ΔCT (sample) – ΔCT (calibrator) (Livak and Schmittgen 2001).

### Immunofluorescence microscopy

G_*α*q/11_ and G_*α*12_ protein expression was localized using immunofluorescence microscopy (IFM) in cryosections of paraformaldehyde‐fixed isolated second‐ and third‐order SMAs from young mice. The freshly dissected SMAs were fixated by submersion in 2% paraformaldehyde for 15 min, followed by cryoprotection via submersion in a 30% sucrose PBS solution for 1–2 h. The vessels were embedded in TissueTek (Sakura Finetek Denmark ApS, Copenhagen, Denmark) and subsequently snap‐frozen in liquid N_2_. We used a Leica CM 1950 cryostat to obtain 5 *μ*m cryosections that were picked up on precleaned microscope slides (Superfrost Plus, Thermo Scientific). The cryosections were stored at −20°C for up to 14 days until used for IFM. Details of the immunostaining protocol were as previously described (Björling et al. 2013). Primary polyclonal antibodies were raised in rabbit against single epitopes from G_*α*q/11_ (sc‐394, Santa Cruz Biotechnology) and G_*α*12_ (ARP54813, Aviva Systems Biology), and were diluted 1:400 and 1:150 in PBS (+0.1% Tween‐20 + 0.1% BSA), respectively. The specificity of the affinity purified anti‐G_*α*q/11_ (sc‐394) antibody was verified in single portal vein myocytes using injection of anti‐*α*q/11 oligonucleotides (Macrez‐Lepretre et al. 1997). The affinity purified anti‐G_*α*12_ antibody (ARP54813) was validated on Western Blot using a cell lysate (human liver) as a positive control (according to the manufacturer's information). Negative control stainings were prepared by pre‐absorption of the primary antibodies for G_*α*q/11_ and G_*α*12_ with their respective antigenic peptides (25 *μ*g/mL), or by omitting the primary antibodies (second Ab only), respectively. The secondary antibody used was Alexa Fluor 594^®^ Donkey‐Anti‐rabbit (diluted 1:800 in PBS/Tween‐20/BSA). Nuclei were stained by treating cryosections with DAPI (DAKO; 1:10,000 dilution). Finally, the sections were covered with Dako antifade reagent and protected with a coverslip. Immunofluorescence was viewed using a BX50 fluorescence microscope (Olympus) equipped with a UPlanApo 40X/NA 1.0 oil immersion quartz objective, and captured using a Retiga 6000 cooled monochrome CCD camera and Q‐Capture Pro7 software (Q‐Imaging Inc., Surrey, BC, Canada).

### Solutions and pharmacological drugs

Krebs’ buffer contained the following (in mmol L^−1^): 118 NaCl, 4.7 KCl, 25 NaHCO_3_, 1.2 KH_2_PO_4_, 1.2 MgSO_4_, 2 CaCl_2_, 5 Glucose. K75 buffer contained the same ion concentrations as Krebs’ buffer except for equimolar substitution of Na^+^ with K^+^. Mounting buffer contained (in mmol L^−1^): 140 NaCl, 5 KCl, 1.2 MgCl_2_, 10 HEPES, 5 Glucose (pH 7.40). Ca^2+^‐free (“Passive diameter”) Krebs’ buffer: 118 NaCl, 4.7 KCl, 25 NaHCO_3_, 1.2 KH_2_PO_4_, 1.2 MgSO_4_, 2 EGTA, 5 Glucose. All solutions containing bicarbonate were equilibrated with 95% O_2_ and 5% CO_2_ to obtain a pH of 7.40 as measured in situ in the myograph bath using a mini pH‐electrode (Metrohm, Switzerland). Stock solutions of pharmacological drugs were prepared in H_2_O or DMSO and stored at −20°C until use (up to 2 months). Krebs’ buffer with final concentrations of the drugs were prepared freshly prior to use, or alternatively the drug was added directly to the myograph bath, while making sure that the solvent concentration did not exceed 0.1%. All drugs were from Sigma‐Aldrich unless otherwise stated. The following drugs and final concentrations were used: AACOCF_3_ (5 *μ*mol L^−1^); BayK 8644 (2 *μ*mol L^−1^; Alomone Labs, Jerusalem, Israel); Bisindolylmaleimide X (BIM‐X) (1 *μ*mol L^−1^); ET‐18‐OCH_3_ (edelfosine) (10 *μ*mol L^−1^); HET0016 (10 *μ*mol L^−1^, Cayman Chemical, Ann Arbor, USA); KD025 (SLx 2119) (5 *μ*mol L^−1^; Cayman Chemical, Ann Arbor, USA); RHC 80267 (20 *μ*mol L^−1^); U‐73122 (0.5 *μ*mol L^−1^); Wortmannin (0.03 *μ*mol L^−1^); YM‐254890 (0.1 *μ*mol L^−1^; Adipogen AG, Liestal, Switzerland; and generous gift from Astellas Pharma, Tokyo, Japan).

### Data analyses and statistics

Myogenic tone (%) at each pressure was calculated as the difference between the passive lumen diameters (*D*_Pas_) and the lumen diameters in the first or second pressure‐diameter curves (D) divided by the respective passive diameters ((*D*_Pas_−*D*)/D_Pas_) × 100). Data are presented as mean ± SEM. A two‐way anova was used to test for statistical differences between myogenic tone curves before vs after an indicated drug treatment. The slope of a linear regression was compared with zero (no slope) to test for age‐dependency in the mRNA expression data as a function of age. A probability level (*P*) lower than 0.05 was considered statistically significant.

## Results

**Figure 1:**
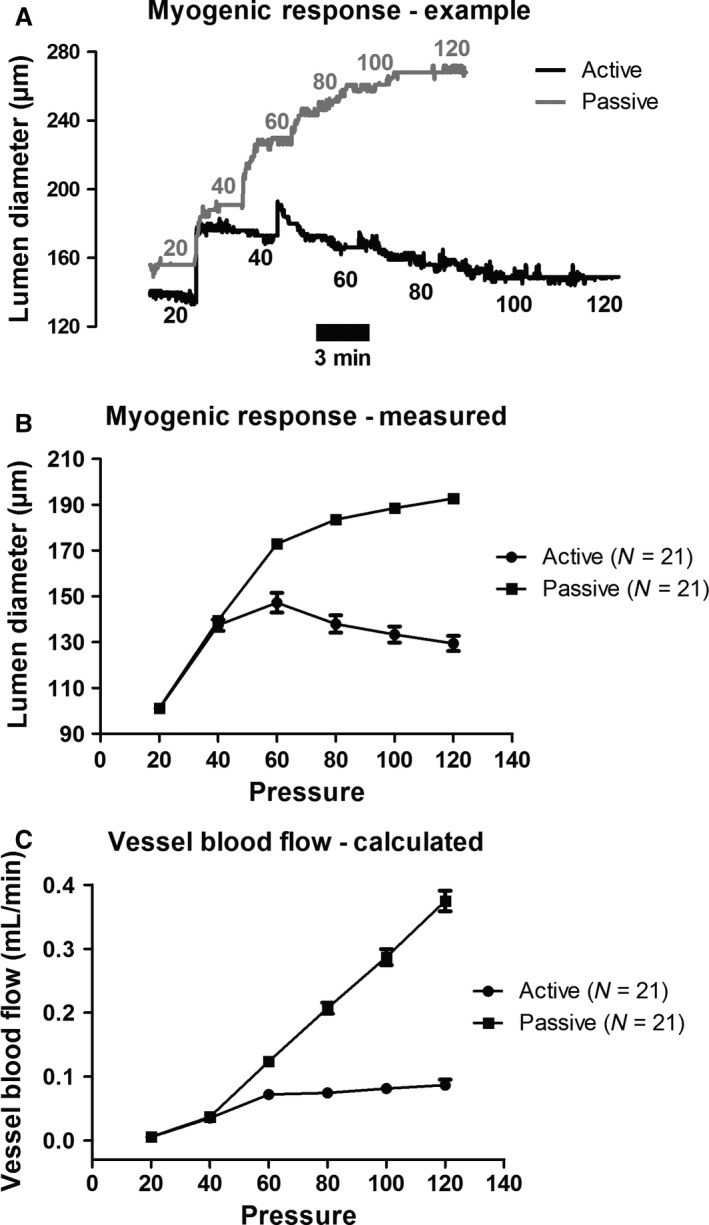
(A) An original trace depicting the active constriction in mouse SMAs when the intraluminal pressure is increased to 60 mm Hg or higher. The passive curve is shown directly above, in which the vessels had been completely relaxed using Ca^2+^‐free Krebs’ solution with the Ca^2+^ chelator EGTA. (B) Measurements of the myogenic response (active) in 21 SMAs from 21 young mice compared to the passive vessel distension in the same vessels. (C) Theoretical single vessel blood flow calculated for both active and passive conditions (as shown in B), assuming vessel length of 1 cm, and using literature values of vascular resistance and blood viscosity (see text for further explanation).

We initially compared the myogenic response (active pressure curve) with the passive distension in Ca^2+^‐chelated buffer (passive curve) in SMA from 21 young male wild type mice (cf. Fig. 1A and B). Since we are utilizing this preparation as a generic model for the myogenic response, we were interested in to what extent the myogenic response in this vascular bed is capable of autoregulating the blood flow. For this purpose, we calculated the theoretical blood flow (pressure range 20–120 mm Hg) in single 1 cm segments of the mesenteric arterial tree using the Hagen‐Poiseuille equation modified with an expression that incorporates relative viscosity (cf. Eqs. (1) and (2)). Our results show that while the blood flow in the passive vessels are increased as expected, the flow in the active vessels is nearly constant in the pressure range from 60 to 120 mm Hg (Fig. 1C). This therefore suggests that, although mouse SMA may not autoregulate intestinal blood flow in vivo, they do have the intrinsic capacity to do so since they are myogenically active when isolated and tested in a pressure myograph.

**Figure 2:**
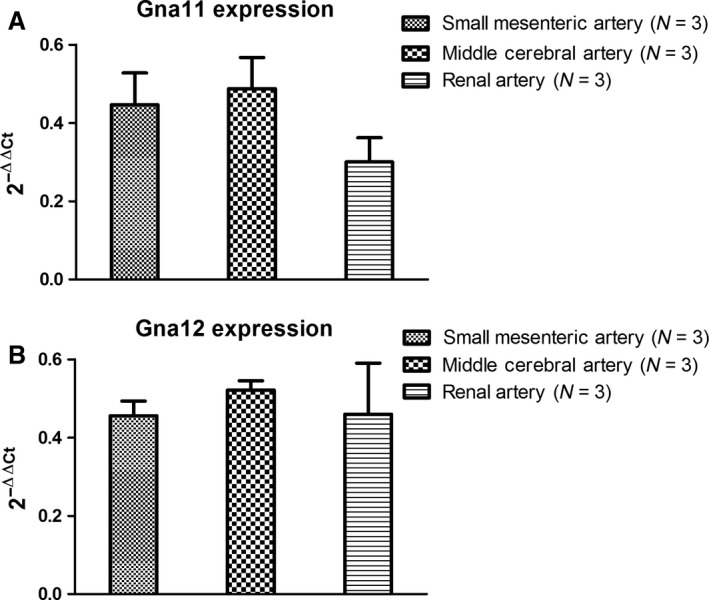
Q‐PCR data (normalized relative ratio) showing mRNA quantitation of the (A) Gna11 gene coding for the G_*α*q/11_ protein, and (B) of the Gna12 gene coding for the G_*α*12_ protein. Data are from three young mice.

**Figure 3:**
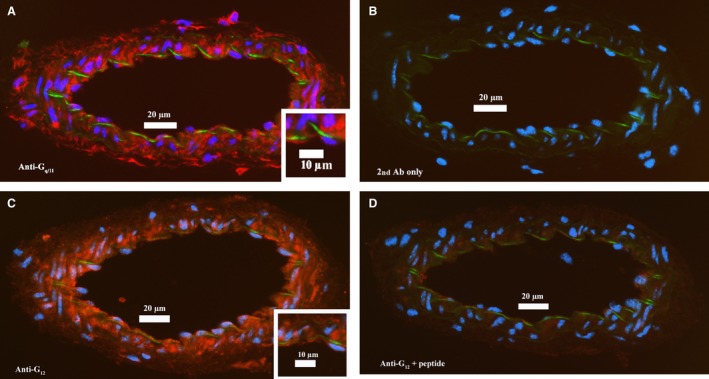
Immunolocalization of G proteins on freeze‐sections of mouse SMAs. (A) Red color showing localization of G_*α*q/11_ protein with abundant staining in *media* (and *intima*) layers, and in a few scattered fibroblasts in the adventitia. Blue color depicts nucleus staining, and a faint green band depicts autofluorescence of the internal elastic laminae (IEL). Inserts in a white frame in both A and C represent an area of each vessel where the positive red staining in the *intima* layer outside the green IEL can be inspected. (B) Absence of positive (red) staining in sections incubated without primary antibody, showing that the secondary antibody does not cross‐react with the mouse sections. (C) Red color showing localization of G_*α*12_ protein with abundant staining in *media* (and *intima*) layers and in a few scattered fibroblasts in the adventitia. See also insert for close‐up of G_*α*12_ staining in the *intima* layer. (D) Strong suppression of red positive staining with anti‐G_*α*12_ antibody preincubated with the peptide sequence used for immunization of rabbits, demonstrating the specificity of this antibody for G_*α*12_. The stainings are representative of the results found in 3 young mice.

Since our hypothesis was that both G_*α*q/11_ and G_*α*12_‐mediated signaling components participate in the generation of spontaneous myogenic tone, we tested whether the expression of both G‐proteins differ at the transcriptional level in MCA, SMA, and in RA from young wildtype mice (Fig. 2A–B). Even though these three vessel types represent myogenically active resistance arteries (MCA and SMA) and conduit arteries (RA) the mRNA expression level of Gna11 and Gna12 did not differ between the vascular beds. Next, we determined the expression and localization of these targets at the protein level in the mesenteric vascular bed using immunostaining with specific antibodies directed against short epitopes of the G_*α*q/11_ and G_*α*12_ proteins (Fig. 3A–D). The abundant immunoreactivity in the media layer of the SMAs in Figure 3A demonstrates expression of G_*α*q/11_ in the VSMCs. Furthermore, a subset of cells in the adventitial layer is also brightly immunoreactive, and we interpret this as being derived from expression in scattered fibroblasts since peripheral nerves would be much smaller. In the enlarged inset in Figure 3A, bright immunostaining is seen in the intima layer on the outside of the internal elastic lamina (the latter shown as a band of green autofluorescence) demonstrating expression of G_*α*q/11_ protein in endothelial cells (ECs) of SMAs. The staining with anti‐G_*α*q/11_ antibody was abolished by pre‐absorbing the antibody with its immunizing peptide (not shown), and the staining was also negative when the primary antibody was omitted (Fig. 3B). Essentially, the same distribution of immunostaining for the anti‐G_*α*12_ antibody is shown in Figure 3C, suggesting that both G_*α*q/11_ and G_*α*12_ are abundantly expressed across VSMCs, ECs, and fibroblasts in the vascular wall of SMAs. The pre‐absorption experiment with the anti‐G_*α*12_ antibody almost completely abolished the immunoreactivity (Fig. 3D), and the staining was also negative when the anti‐G_*α*12_ antibody was omitted (not shown).

**Figure 4:**
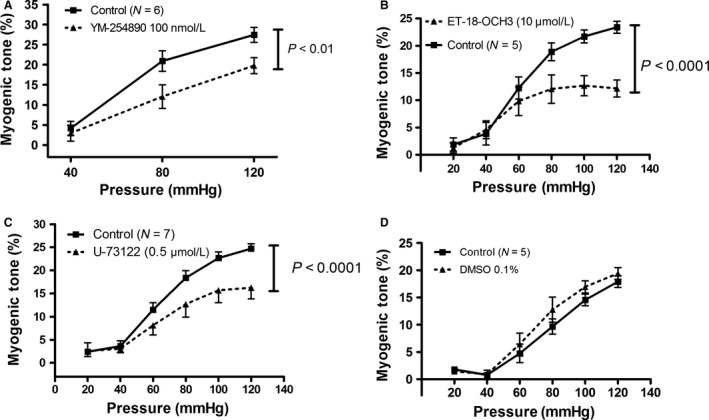
(A) Effect of specific G_*α*q/11_ inhibitor YM‐254890 (100 nmol L^−1^) on myogenic tone development in mouse SMAs. (B) Effect of PI‐PLC inhibitor ET‐18‐OCH3 (10 *μ*mol L^−1^; Edelfosine) on MT. (C) Effect of general PLC inhibitor U‐73122 (0.5 *μ*mol L^−1^) on MT. (D) Lack of effect of vehicle DMSO (0.1%) on MT. All drugs were used at concentrations that did not inhibit KCl‐induced constriction when tested in the same SMAs. N is equal to the number of SMAs tested in the same number of young mice.

In the following experiments, we tested the effects of inhibition of G_*α*q/11_ and its downstream signaling components on myogenic tone development in SMAs from young mice. The drugs were all used at concentrations that did not inhibit the peak of the KCl‐induced vasoconstrictions. First, we used a low concentration (100 nmol L^−1^) of the specific G_*α*q/11_ inhibitor YM‐254890, which caused a significant reduction in MT but did not abolish it (Fig. 4A). We could not increase the concentration of YM‐254890, while inferring a specific effect on the myogenic response, since higher concentrations blunted the KCl‐induced constrictions. Next, we tested the maximal effects of two structurally different Phosholipase C (PLC) inhibitors, ET‐18‐OCH3 and U‐73122. Both of these PLC inhibitors induced a highly significant inhibition of MT without affecting the KCl‐induced constriction (Fig. 4B–C). However, U‐73122 could only be tested at a low concentration (0.5 *μ*mol L^−1^) to avoid effects on the KCl responses. There were no significant effects of vehicle DMSO (0.1%) on MT in SMAs from young mice (Fig. 4D).

**Figure 5:**
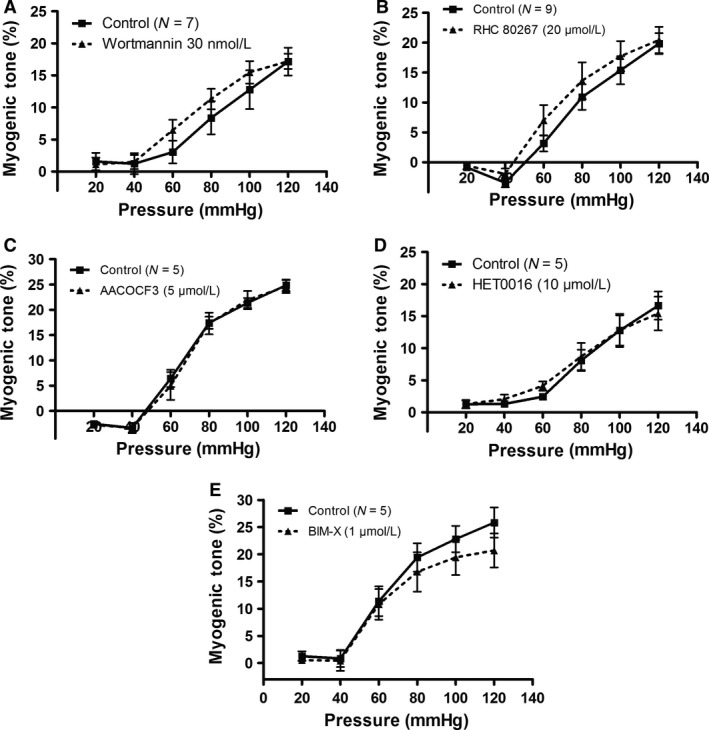
(A) Lack of effect of the PI3‐Kinase inhibitor Wortmannin (30 nmol L^−1^) on MT in mouse SMAs. (B) Lack of effect of diacylglycerol lipase (DAG) inhibition with RHC 80267 (20 *μ*mol L^−1^) on MT in SMAs. (C) Lack of effect of Phospholipase A_2_ (PLA_2_) inhibition with AACOCF3 (5 *μ*mol L^−1^) on MT in SMAs. (D) Lack of effect of selective inhibition of 20‐HETE with HET0016 (10 *μ*mol L^−1^) on MT in SMAs. (E) Lack of effect of PKC inhibitor BIM‐X (1 *μ*mol L^−1^) on MT in SMAs. Drugs were used at concentrations that did not inhibit KCl‐induced constriction when tested in the same SMAs. N is equal to the number of SMAs tested in the same number of young mice.

We next investigated the role of signaling components downstream of G_*α*q/11_ and PLC, namely IP_3_ and diacylglycerol (DAG). We first used an inhibitor of PI3‐kinase (30 nmol L^−1^ Wortmannin) and DAG lipase (20 *μ*mol L^−1^ RHC 80267), but there were no effects on MT of these agents at concentrations that did not block KCl‐responses (Fig. 5A–B). Using higher Wortmannin concentrations did not only block MT, but it also blocked the KCl‐responses (data not shown) most likely via a direct inhibition of MLCK (Nakanishi et al. 1992). Then, we questioned whether the cascade leading from arachidonic acid release to 20‐HETE production would be important. However, the Phospholipase A_2_ inhibitor AACOCF3 (5 *μ*mol L^−1^) and the 20‐HETE production (via *ω*‐hydroxylation) inhibitor HET0016 (10 *μ*mol L^−1^) did not have any effects (Fig. 5C–D). In preliminary experiments (*N* = 2) we tested a higher HET0016 concentration (30 *μ*mol L^−1^, as well as the effect of 10 *μ*mol L^−1^ HET0016 on MT induced by a pressure step from 60 to 100 mm Hg in SMAs preconstricted using 2 nmol L^−1^ NPY + 100 nmol L^−1^ NE. However, neither of these HET0016 incubation protocols had any effects on MT, contrary to previous effects of 10 *μ*mol L^−1^ HET0016 shown in rat mesenteric arteries (Inoue et al. 2009). The PKC inhibitor BIM‐X at a concentration (1 *μ*mol L^−1^) previously demonstrated to specifically inhibit PKC (Jover et al. 1999) did not have a significant effect on MT (Fig. 5E), and similar results were obtained in preliminary experiments using a structurally different PKC inhibitor Calphostin C (10–100 nmol L^−1^; *N* = 2; data not shown). The lack of a role of PKC prompted us to investigate the role of the Rho‐kinase pathway in the myogenic response.

**Figure 6:**
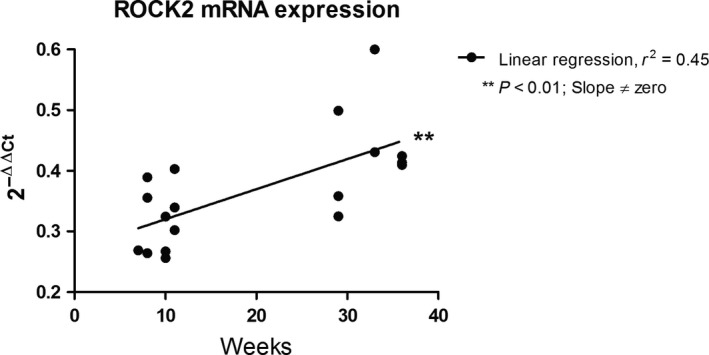
ROCK2 mRNA expression in SMAs measured by quantitative real‐time PCR and plotted as a function of the age of the mice (*N* = 18). A positive slope significantly different from zero indicates age‐dependent increase in ROCK2 expression.

**Figure 7:**
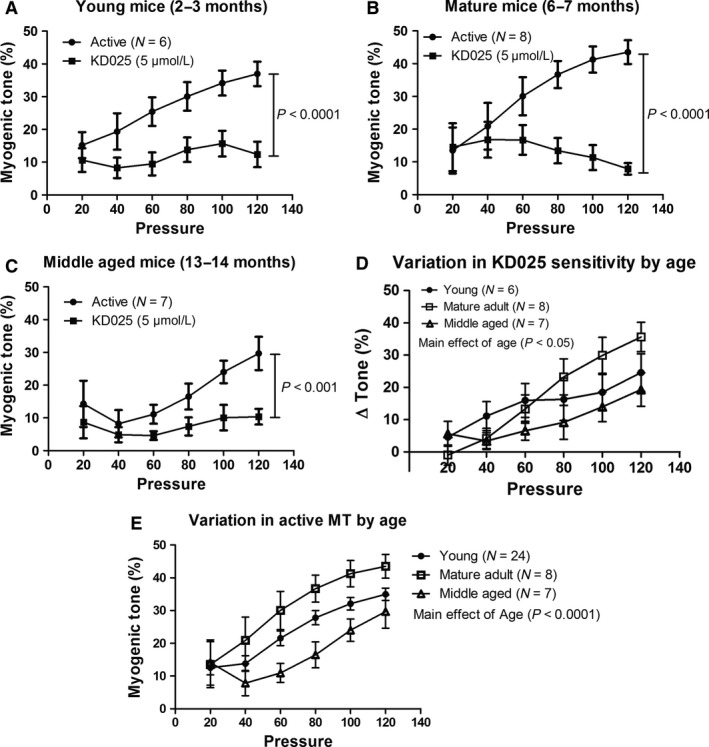
(A) Effect of specific ROCK2 inhibitor KD025 (5 *μ*mol L^−1^) on MT in SMAs from young mice (2–3 months old). A total of six vessels from five mice were tested. (B) Effect of KD025 (5 *μ*mol L^−1^) on MT in SMAs from mature adult mice (6–7 months old). A total of eight vessels from five mice were tested. (C) Effect of KD025 (5 *μ*mol L^−1^) on MT in SMAs from middle aged mice (13–14 months old). A total of seven vessels from three mice were tested. (D) Significant age‐dependent variation (main effect) in KD025 sensitivity of MT development (data calculated from the experiments shown in A–C). (E) Significant age‐dependent variation (main effect) in active MT from young (*N* = 24), mature adult (*N* = 8), and middle aged mice (*N* = 7).

**Figure 8:**
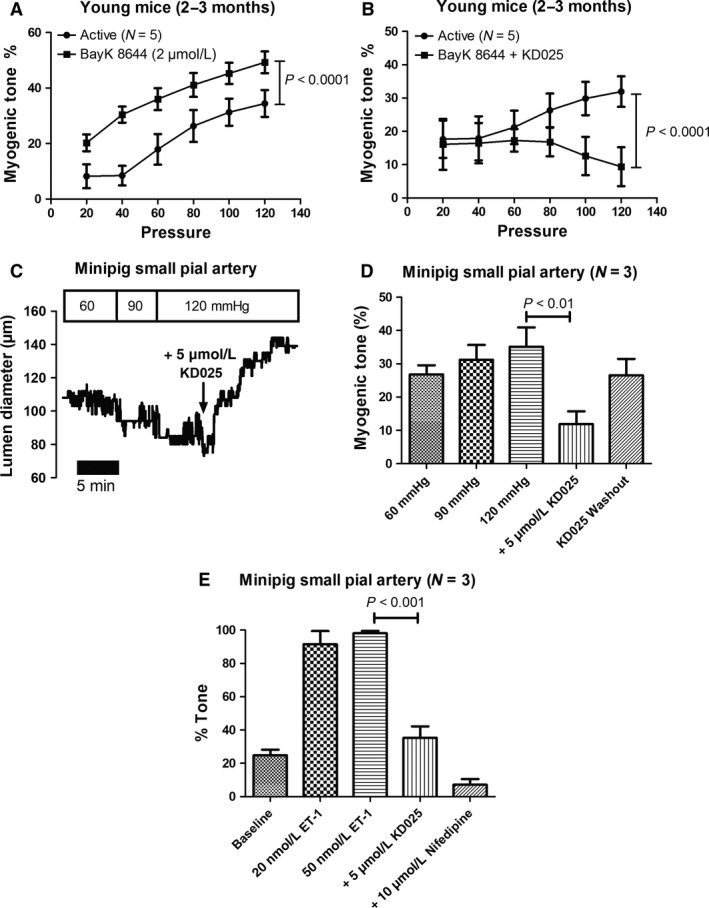
(A) Effect of the L‐type Ca^2+^ channel agonist BayK 8644 (2 *μ*mol L^−1^) on MT. A total of five vessels from five young mice were tested. (B) In a parallel series of experiment the effect of the ROCK2 inhibitor KD025 (5 *μ*mol L^−1^) in the presence of BayK 8644 was investigated. A total of five vessels from four young mice were tested. (C) For comparison, the effect of ROCK2 inhibition with KD025 (5 *μ*mol L^−1^) was tested in a small pial arteries from three healthy Göttingen minipigs. This original trace showing a pial vessel that develops MT at 60, 90 and 120 mm Hg, and MT is strongly inhibited by direct bath application of KD025. Note that the full effect of KD025 takes 5–10 min to be established. (D) The same experiment but with results for all three minipigs depicted as myogenic tone. Also shown is the effect of washout (15–20 min) of the drug. (E) Active vasoconstriction to direct bath application of ET‐1 (20 and 50 nmol L^−1^) in small pial arteries from three minipigs pressurized at 60 mm Hg. Potent inhibition of vascular tone is induced by KD025, and the remainder of tone is abolished by direct bath application of the L‐type antagonist nifedipine (10 *μ*mol L^−1^).

In a previous study of MT in mouse SMAs, we noted that the magnitude of MT increased by age (Mikkelsen et al. 2016). The RhoA/Rho‐kinase pathway can be activated by both G_*α*12_ and G_*α*q/11_ proteins (Vogt et al. 2003; Lutz et al. 2005). As Rho‐kinase is crucially involved in the myogenic response in rat middle cerebral arteries (Gokina et al. 2005; Jarajapu and Knot 2005) and skeletal muscle resistance arteries (Bolz et al. 2003), and since it is the ROCK2 isoform that is involved in VSMC contractility (Wang et al. 2009), we evaluated the role of ROCK2 in the aging‐induced increases in MT. We first questioned whether the expression level of ROCK2 was increased in SMAs from mature adult mice (33 ± 1 weeks; *N* = 8) compared to young mice (9 ± 0.5 weeks; *N* = 10). As shown in Figure 6, the mRNA expression of ROCK2 increased significantly (*P* < 0.01) as a function of age in SMAs from mice in the age range tested. We next tested the effect of the ROCK2 inhibitor KD025 (IC_50_ = 105 nmol L^−1^), which has a 200‐fold selectivity over ROCK1 (IC_50_ = 24 *μ*mol L^−1^) (Boerma et al. 2008). We found that a concentration of 5 *μ*mol L^−1^ KD025 did not inhibit the peak of the KCl‐induced constrictions (30 sec after stimulation), but it did inhibit the sustained constrictions (3 min after stimulation; see appendix Fig. S1). The KD025 effects were evaluated on MT in SMAs from mice of three different age groups: 2–3 months (young), 6–7 months (mature adult), and 13–14 months (middle aged). There was a highly significant effect of 5 *μ*mol L^−1^ KD025 in all three age groups (Fig. 7A–C), and by inspecting the MT curves before and after KD025 addition this effect seemed largest in the SMAs from mature adult mice. When we evaluated the KD025 sensitivity using the difference in MT before vs after KD025 addition (Δtone; Fig. 7D), there was a significant variation in KD025 sensitivity attributed to age, which confirms our initial observation. When we compared the MT under control conditions (active) across age groups, there was a highly significant variation in MT attributed to the age, but surprisingly, the MT was apparently highest in the mature adult group, and lowest MT in the middle aged mice (Fig. 8E). Vehicle DMSO (0.1%) was at this point also tested in middle aged mice, but there were no significant effects on MT (*N* = 3; data not shown).

Having established an age‐dependent role of ROCK‐mediated MT in SMAs from mice, we next questioned the relative importance of ROCK2 vs other signaling molecules, and in other species. As MT in mouse SMAs can both be abolished by inhibiting the voltage‐dependent Ca^2+^ influx via L‐ and T‐type channels (Björling et al. 2013) and by inhibiting the ROCK‐mediated Ca^2+^ sensitivity (present study), we speculated that there could be an interaction between these two pathways proximal to their effects on MLCK and MLCP, respectively. Such effects could hypothetically be mediated via Ca^2+^‐dependent stimulation of RhoGEFs or RhoA activity (Guilluy et al. 2010; Momotani and Somlyo 2012). To shed light on this possibility, we investigated the effects of stimulating Ca^2+^ influx via L‐type channels using Bay K8644 in the absence or presence of the ROCK2 inhibitor KD025. Our rationale was that if ROCK2 activation is the only way through which Ca^2+^ sensitivity can be increased during development of sustained MT, then we should see an abolished MT to KD025 even in the presence of the L‐type channel agonist Bay K8644. The effect of addition of 2 *μ*mol L^−1^ Bay K8644 alone was a highly significant increase in MT throughout the pressure range tested in SMAs from young mice (Fig. 8A). In parallel experiments where the SMAs were incubated with 2 *μ*mol L^−1^ Bay K8644 + 5 *μ*mol L^−1^ KD025 we observed a highly significant inhibition of MT, which manifested in the pressure range from 80 to 120 mm Hg (Fig. 8B). This suggested that ROCK2 is a crucial determinant of MT in the pressure range important for blood flow autoregulation and cannot be bisected by other Ca^2+^‐stimulated mechanisms. Finally, to strengthen the translational aspects of our study, we were able to test the effect of KD025 on small pial arteries isolated from the brains of three healthy female Göttingen minipigs. Inhibition of ROCK2 by 5 *μ*mol L^−1^ KD025 caused robust inhibition of the MT (at 120 mm Hg; Fig. 8C–D) and of the tone induced by exposure to the vasoactive agonist endothelin‐1 (ET‐1; 50 nmol L^−1^; Fig. 8E). The effect of KD025 was partially reversible and did not completely abolish MT or ET‐1 induced constriction in the minipig pial vessels (Fig. 8D–E). Further addition of the L‐type antagonist nifedipine (10 *μ*mol L^−1^) abolished the remaining tone after blocking ET 1‐induced tone by KD025 (Fig. 8E).

## Discussion

### Relevance of myogenic tone in mouse small mesenteric arteries

The myogenic response is crucial for blood flow autoregulation in the brain, heart, and kidney, and it determines the basal tone in several other systemic vascular beds, such as that of the skeletal muscle. As the small intestinal circulation has a relatively high flow during rest and digest, whereas the blood flow is shunted away from this vascular bed during flight or fight syndrome by a large increase in sympathetic output, the question is to what extent a myogenic response in the mesenteric arcade arteries and mucosal resistance vessels contributes to the regulation of blood flow to the small intestine. If autoregulation is present in the intestine, it is more often thought of as the result of autoregulatory “escape” in which local metabolic factors inhibit sympathetic tone (Granger et al. 1980). Nevertheless, small mesenteric arteries in rats (fourth to fifth order branches) and mice (second to third order branches) have in several studies been shown to harbor a myogenic response when isolated and tested in a pressure myograph (Bund 2001; Scotland et al. 2001; Gros et al. 2002). For this reason, we evaluated the theoretical blood flow emanating from the myogenic response that was measured in a large group of young mice, and we found that the mouse mesenteric arcade arteries (second to third order) are spontaneously myogenically active and capable of autoregulating the vessel blood flow in a physiologically relevant pressure range from 60 to 120 mm Hg (cf. Fig. 1A–C). Since the mouse SMAs are often used to characterize arterial function in knock‐out and disease models, our data showing the relevance of the myogenic response in this vascular bed is potentially important.

### Role of G protein‐mediated signaling in myogenic tone

We found that the Gna11 and Gna12 genes encoding the G_*α*q/11_ and G_*α*12_ proteins were abundantly expressed in cerebral, renal and mesenteric arteries from wild type mice. Furthermore, immunolocalization in mouse SMAs showed abundant expression of G_*α*q/11_ and G_*α*12_ proteins in both VSMCs, ECs, and in adventitial cells presumed to be fibroblasts. We tested the G_*α*q/11_‐specific inhibitor YM‐254890, which inhibits the GDP/GTP‐exchange of the receptor *α*‐subunit by inhibiting its release of GDP (Takasaki et al. 2004; Nishimura et al. 2010), and found that it very significantly inhibited approximately 30–40% of the myogenic tone in SMAs from young mice. Thus, it is clear that the G_*α*q/11_ protein is expressed and involved in spontaneous MT development. Due to the lack of a G_*α*12_‐specific pharmacological blocker, we were not able to test the relative contribution of G_*α*12_ proteins to MT development, but we have addressed the role of the G_*α*12_/RhoA/ROCK pathway by investigating the impact of ROCK2 inhibition (see Discussion below).

It has been known for several years that intracellular signaling components downstream of G protein‐coupled receptor activation are involved in the myogenic response mechanism. First, PLC contributes to the myogenic mechanism in rat small cerebral arteries (Osol et al. 1993; Jarajapu and Knot 2002; Gonzales et al. 2014), rat and hamster cremaster muscle arterioles (Bakker et al. 1999; Jackson and Boerman 2017), rat ophthalmic artery (Ito et al. 2007), and human subcutaneous resistance arteries (Coats et al. 2001). Elevation of transmural pressure in isolated dog renal arteries elicited production of both IP_3_ and DAG consistent with a pressure‐dependent activation of PLC (Narayanan et al. 1994). However, in Hamster cheek pouch arterioles no role was found for PLC in MT (Jackson and Boerman 2017). Addition of the membrane‐permeable DAG analogue OAG did not change MT in rat cremaster muscle arterioles (Hong et al. 2016) or human subcutaneous resistance arteries (Coats et al. 2001), whereas inhibition of DAG lipase abolished MT in human subcutaneous resistance arteries (Coats et al. 2001). Our present results with mouse SMAs confirm a role of PLC in MT (Fig. 4B–C), but does not support a role of PI3 kinase or DAG lipase, which regulate the turnover of IP_3_ and DAG in the VSMCs, respectively (Fig. 5A–B). Interestingly, a recent study found that it was the Phosphatidylcholine‐specific PLC (PC‐PLC) and not Phosphoinositide‐PLC (PI‐PLC) that mediated MT in mouse mesenteric arteries (Mauban et al. 2015). To this end, we have used a general PLC inhibitor (U73122) as well as a PI‐specific inhibitor (ET‐18‐OCH3/edelfosine), which both inhibited myogenic tone at concentrations that did not block KCl‐induced vasoconstriction. The discrepancy between the PI‐PLC vs PC‐PLC involvement might be due to a modulatory role of the endothelium since the latter study (Mauban et al. 2015) used continuous intraluminal flow of mouse SMAs during the myogenic tone measurements, whereas we used no‐flow conditions to eliminate activation of shear stress‐dependent responses.

DAG lipase mediates the conversion of DAG to arachidonic acid (AA). 20‐HETE, the *ω*‐hydroxylation product of AA, has been shown to participate in myogenic tone development in rat middle cerebral arteries (Gebremedhin et al. 2000), in renal afferent arterioles from mouse, rat, and rabbit (Imig et al. 1994; Ge et al. 2013), and in rat mesenteric arteries preconstricted by an *α*_1_‐agonist and neuropeptide Y (Inoue et al. 2009). However, our present results indicated that the pathway leading from Phospholipase A_2_ (PLA_2_) and/or DAG lipase to AA and 20‐HETE production does not contribute to MT development in mouse SMAs (Fig. 5B–D).

Myogenic tone development downstream of DAG production may also occur by activation of PKC as shown in rat cerebral arteries (Osol et al. 1991; Jarajapu and Knot 2005), rat cremaster muscle arterioles (Bakker et al. 1999), mouse mesenteric arteries (Lagaud et al. 2001), and in human subcutaneous resistance arteries (Coats et al. 2001). However, the contribution of PKC to MT was minor compared to Rho‐kinase in rat middle cerebral arteries (Jarajapu and Knot 2005) and was not present at all in rat ophthalmic arteries (Ito et al. 2006). We could not confirm a role of PKC in MT in this study using the PKC inhibitor BIM‐X, which is in agreement with the two latter studies, but in disagreement with a previous investigation of MT in mouse SMAs, where the PKC inhibitors Calphostin C and LY333531 (a PKC_*β*_‐specific inhibitor) were used (Lagaud et al. 2001). The discrepant results obtained may be due to the employment of different PKC antagonists and concentrations, vascular beds or age of the animals. The depolarization downstream of PLC activation was not further investigated, but according to previous studies it may involve activation of TRPC6 channels (Welsh et al. 2002; Inoue et al. 2009; Gonzales et al. 2014), TRPM4 channels (Gonzales et al. 2014) or inhibition of a “leaky” K^+^ channel sensitive to the nonspecific K_V_ channel blocker XE‐991 (Schleifenbaum et al. 2014). However, the involvement of TRPC6 channels downstream of pressure‐dependent activation of GPCRs must be interpreted with caution since mice deficient in TRPC6 channels did not show an impaired myogenic tone in SMAs (Schleifenbaum et al. 2014).

### Role of ROCK2 and aging in myogenic tone

We hypothesized that ROCK2, which is the predominant Rho‐kinase isoform involved in VSMC contractility (Wang et al. 2009), is directly involved in the aging‐induced increase in myogenic tone as previously found by us (Mikkelsen et al. 2016). When we investigated the ROCK2 mRNA expression level in SMAs from mice ranging in age from 2 to 9 months, we found a very significant positive correlation between age and ROCK2 expression (Fig. 6). In the subsequent testing of the ROCK2‐specific inhibitor KD025 (SLx 2119), we included an additional group of mice that was 13–14 months old. The myogenic tone was highly significantly inhibited in all three age groups (Fig. 7A–C), but by comparing the KD025 sensitivity between these three age groups we found a significant age‐dependent variation (Fig. 7D), which is in agreement with the age‐dependent increase in ROCK2 expression. Furthermore, this is supported by the observation that there was a significant age‐dependent variation in the total active MT when comparing the three age groups (Fig. 7E). However, it was apparent that the mature adult mice had a higher KD025 sensitivity and a higher active MT than the young and middle aged mice, respectively, which may at first seem counterintuitive. Nevertheless, previous studies have found an age‐related decline in MT in both mesenteric arteries (Gros et al. 2002) and middle cerebral arteries (Springo et al. 2015) when comparing adult mice of 3–6 months of age with old mice at about 12–24 months of age. Thus, it seems that male mice at mature adulthood have a peak in their basal myogenic tone, which could be related to a lower estrogen‐to‐androgen ratio in this age group compared to younger as well as older male mice, and compared to age‐matching female mice (Geary et al. 2000a,b; Chan et al. 2012). These results suggest that the increased susceptibility to hypertension and cardiovascular disorders at middle age might be related to an increased ROCK2‐dependent basal myogenic tone, which is in agreement with a recent study showing an age‐dependent increase in hormonal activation of G_*α*q/11_‐ and G_*α*12_‐mediated vasoconstriction and increased blood pressure (Wirth et al. 2016).

Previous studies have shown that the sustained contraction of rat caudal arteries to high‐KCl was inhibited by the ROCK1/2 inhibitor Y‐27632, an effect ascribed to a Ca^2+^‐dependent activation of RhoA involving genistein‐sensitive tyrosine phosphorylation (Mita et al. 2002, 2013). Using the ROCK2 inhibitor KD025 we can confirm this inhibition of the sustained KCl‐induced constriction (plateau) in mouse SMAs (Fig. S1). However, since KD025 did not inhibit the initial KCl‐induced constriction (peak), we argue that there were no unspecific effects of KD025 in this study. Rho‐kinase is capable of phosphorylating many targets, and among these are ion channels involved in vascular tone regulation. A recent study suggested that ROCK participates in myogenic tone development in rat cerebral parenchymal arterioles by activating TRPM4 channels, leading to Na^+^ influx, depolarization, voltage‐dependent Ca^2+^ influx, and cross‐bridge cycling (Li and Brayden 2017). This model, however, does not account for the substantial increase in Ca^2+^ sensitivity known to occur during MT development (Lagaud et al. 2002; Matchkov et al. 2002; Schubert et al. 2008). Regulation of G_*α*q/11_‐ and G_*α*12_‐mediated signaling through RhoA is achieved by RhoGEFs, see reviews by (Siehler 2009; Momotani and Somlyo 2012; Althoff and Offermanns 2015). It has been demonstrated that G_*α*12_ activates RhoA through p115RhoGEF, PDZ‐RhoGEF, and LARG (Meyer et al. 2008; Wirth et al. 2008; Artamonov et al. 2013). G_*α*q/11_ can activate RhoA via p63RhoGEF and LARG (Booden et al. 2002; Vogt et al. 2003; Lutz et al. 2005; Momotani et al. 2011). Furthermore, p115RhoGEF is activated by a PLC/IP_3_/Ca^2+^/Jak2‐dependent mechanism downstream of G_*α*q/11_ activation (Guilluy et al. 2010). It must be mentioned here that RhoA can also be activated directly by mechanical forces acting on cells and tissues (Lessey et al. 2012), but this occurs at a timescale of 5–10 min (Zhao et al. 2007) that is slower than activation of the myogenic response. Several key enzymes in vascular tone regulation are Ca^2+^‐dependent (e.g., Cl_Ca_, PLC, PKC, PLA_2_, RhoGEFs), and we therefore aimed to establish how important ROCK2 is in MT development compared to upstream Ca^2+^‐activated signaling mechanisms. To do so, we tested the effect on MT of increasing the cytosolic Ca^2+^ concentration pharmacologically using an agonist of the L‐type calcium channels in the presence or absence of the ROCK2 inhibitor. We found that, by inhibiting ROCK2, the Ca^2+^‐induced increase in MT was abolished at pressures higher than 60 mm Hg, and we therefore conclude that there are no other major calcium‐dependent signaling mechanisms that can replace ROCK2 in controlling the ratio between MLCK and MLCP activities in VSMCs during pressure‐induced vasoconstriction.

Nonselective inhibition of both ROCK isoforms by Fasudil reduces cerebral vasospasms following subarachnoid hemorrhage (Shimokawa and Takeshita 2005; Budzyn et al. 2006), and reduces forearm resistance in patients suffering from essential hypertension (Masumoto et al. 2001) and heart failure (Kishi et al. 2005). Our results suggest that modulating ROCK2 is a potentially important strategy to develop treatment for disorders that are caused by disturbances in microcirculatory blood flow. A recent Phase 1 study in healthy human volunteers concluded that KD025 is “orally available, and well tolerated, without significant adverse events related to treatment with the drug” (Zanin‐Zhorov et al. 2014), which makes it a future potential drug candidate to manipulate pathophysiological changes in resistance artery tone in patients. However, before testing it further in relation to human microvascular tone disturbances, it would be important to translate our findings in mice to a large animal model with higher degree of similarity with the human cardiovascular system. The Göttingen minipig is a translational biomedical model, and we performed additional experiments on small pial arteries from the brain of healthy Göttingen minipigs. The porcine small pial arteries developed substantial myogenic tone at pressures from 60 to 120 mm Hg, and constricted potently to endothelin‐1 (20–50 nmol L^−1^ ET‐1). When the ROCK2 inhibitor KD025 was applied at the same concentration as used in our experiments with mouse SMAs, the myogenic tone and ET‐1 induced vasoconstriction was potently inhibited. This result demonstrates that it is feasible to test the role of ROCK2 in the cerebral vasculature of Göttingen minipigs, and suggests that its crucial role in MT development in mice can be translated to larger animal species having a higher degree of similarity with the human cardiovascular system.

**Figure 9:**
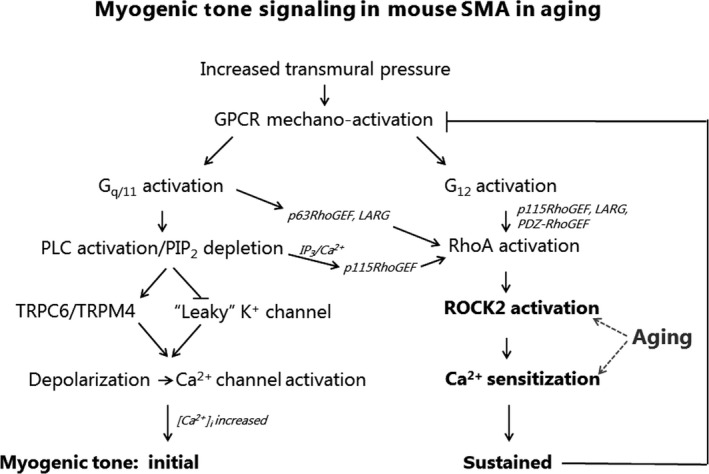
Flowchart depicting our current model of the G protein‐mediated signaling events leading to age‐dependent myogenic tone development in mouse small mesenteric arteries. The left side of the graph shows the events leading to the initial/rapid MT development, and the right side shows events responsible for the sustained MT development. Arrows indicate stimulation of a process or molecule, and inhibition or negative feedback is shown by a line with a perpendicular bar. ROCK2 activation/Ca^2+^ sensitization are highlighted due to their importance for the age‐dependent effects shown in this study. See text for further explanations.

To summarize the signaling mechanism proposed in our discussion and depicted in Figure 9, an increase in wall stress activates a GPCR, which leads to activation of both G_*α*q/11_ and G_*α*12_ proteins. PLC activation causes PIP_2_ depletion from the plasma membrane, which may inhibit a background (“leaky”) K^+^ conductance. TRPC6 and/or TRPM4 channels may also be activated downstream of G_*α*q/11_ and PLC activation. Both TRP channel activation and K^+^ channel inhibition leads to myogenic depolarization, which stimulates voltage‐dependent Ca^2+^ entry leading to MLCK activation and cross‐bridge cycling in VSMCs. In parallel, G_*α*q/11_ and G_*α*12_ activation promotes RhoGEF/RhoA‐dependent activation of ROCK2, which inhibits MLCP and leads to Ca^2+^ sensitization with sustained contraction of VSMCs. Sustained MT leads to normalization of wall stress, which provides negative feedback on the mechanical activation of GPCRs. In mature adult mice, the increased sustained MT in SMAs is caused by an age‐dependent increase in ROCK2 expression and activity, which leads to an increased Ca^2+^ sensitization. ROCK2 activation is an obligatory step in sustained MT development. The proposed signaling mechanisms might be part of a chain of events leading to elevated blood pressure at an advanced age.

## Conflict of Interest

JLH is an employee and shareholder of Novo Nordisk A/S, Måløv, Denmark. TPL is an employee of Novo `Nordisk A/S, Måløv, Denmark.

## Supporting information




**Figure S1.** The vasoconstrictor response to high‐KCl (K75) was measured before and after addition of 5 μmol L^−1^ KD025 in young (A), mature adult (B), and middle aged mice (C).
