# Supplementary material for: Role of age, Rho‐kinase 2 expression, and G protein‐mediated signaling in the myogenic response in mouse small mesenteric arteries
Source: Physiol Rep. 2018 Sep 10;6(17):e13863. doi: 10.14814/phy2.13863 (PMC6129776; doi:10.14814/phy2.13863)
Supplement: Supplementary file 1 — Figure S1. The vasoconstrictor response to high‐KCl (K75) was measured before and after addition of 5 μmol L−1 KD025 in young (A), mature adult (B), and middle aged mice (C). [file PHY2-6-e13863-s001.docx]

## Supporting Information

**Fig. S1:**


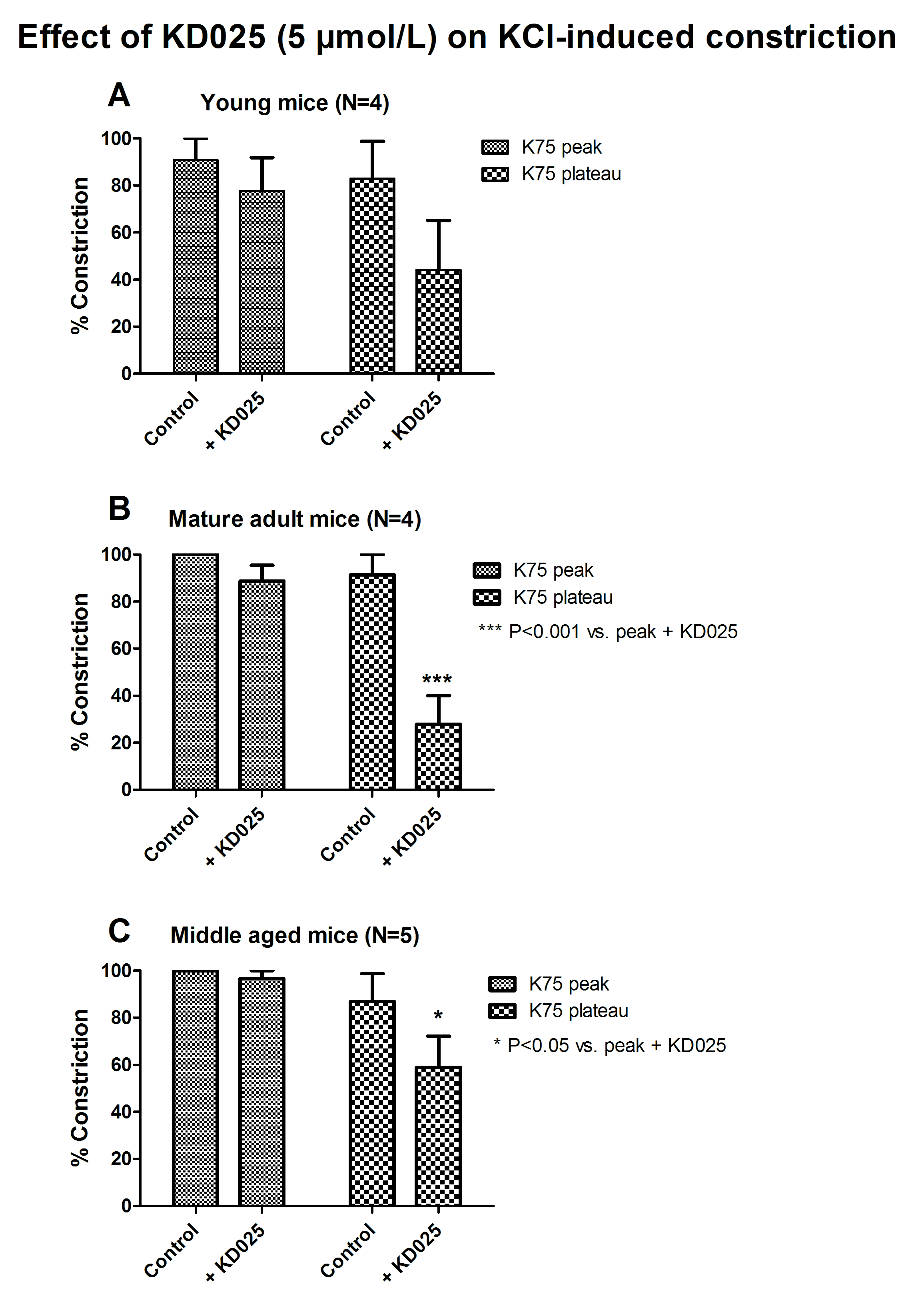


Figure S1: The vasoconstrictor response to high-KCl (K75) was measured before and after addition of 5 µmol/L KD025 in young (A), mature adult (B), and middle aged mice (C). The constrictor responses were evaluated 30 sec after introducing high-KCl (peak constriction) and 3 min after when the constriction was at steady state (plateau constriction). There were no differences between the peak responses with or without KD025. There was an effect of KD025 on the High-KCl plateau responses, as previously described for the effect of the ROCK1/2 inhibitor Y-27632 in rat tail artery (1). See Discussion for further explanation.

1. **Mita M, Yanagihara H, Hishinuma S, Saito M and Walsh MP. Membrane depolarization-induced contraction of rat caudal arterial smooth muscle involves Rho-associated kinase. *Biochem J* 364: 431-440, 2002.**
